# Molecular Detection of Selected Pathogens with Zoonotic Potential in Deer Keds (*Lipoptena fortisetosa*)

**DOI:** 10.3390/pathogens10030324

**Published:** 2021-03-10

**Authors:** Remigiusz Gałęcki, Jerzy Jaroszewski, Tadeusz Bakuła, Eloiza M. Galon, Xuenan Xuan

**Affiliations:** 1 Department of Veterinary Prevention and Feed Hygiene, Faculty of Veterinary Medicine, University of Warmia and Mazury in Olsztyn, 10-719 Olsztyn, Poland; bakta@uwm.edu.pl; 2 Department of Pharmacology and Toxicology, Faculty of Veterinary Medicine, University of Warmia and Mazury in Olsztyn, 10-719 Olsztyn, Poland; jerzyj@uwm.edu.pl; 3 National Research Center for Protozoan Diseases, Obihiro University of Agriculture and Veterinary Medicine, Obihiro 080-8555, Japan; eloizagalon@gmail.com (E.M.G.); gen@obihiro.ac.jp (X.X.)

**Keywords:** deer keds, ectoparasite, Hippoboscidae, louse flies, PCR, vector, infectious diseases, vector-borne diseases

## Abstract

Deer keds are obligatory hematophagous ectoparasites of birds and mammals. Cervids serve as specific hosts for these insects. However, ked infestations have been observed in non-specific hosts, including humans, companion animals, and livestock. *Lipoptena fortisetosa* is a weakly studied ectoparasite, but there is evidence to indicate that it continues to spread across Europe. The existing knowledge on deer keds’ impact on wildlife is superficial, and their veterinary importance is enigmatic. *Lipoptena fortisetosa* is a species with vectorial capacity, but potential pathogen transmission has not been assessed. The objective of this study was to evaluate the prevalence of selected pathogens in *L. fortisetosa* collected from cervids and host-seeking individuals in the environment. Out of 500 acquired samples, 307 (61.4%) had genetic material from at least one tested pathogen. Our research suggests that *L. fortisetosa* may be a potential vector of several pathogens, including *A. phagocytophilum*, *Babesia* spp., *Bartonella* spp., *Borellia* spp., *Coxiella*-like endosymbionts, *Francisiella tularensis*, *Mycoplasma* spp., *Rickettsia* spp., and *Theileria* spp.; however, further, more extensive investigations are required to confirm this. The results of the study indicate that keds can be used as biological markers for investigating the prevalence of vector-borne diseases in the population of free-ranging cervids.

## 1. Introduction

The role of vectors in the transmission of infectious diseases has been researched extensively in recent years. The main arthropods with vector capacity are ticks, mosquitos, black flies, fleas and lice. Many indigenous insects with vector capacity have not been thoroughly investigated in Europe [1,2,3]. One of the most underestimated ectoparasites is the deer ked.

Deer keds (syn. louse flies) of the genus Lipoptena (family Hippoboscidae) are potential vectors of infectious diseases that have not been thoroughly studied to date [4]. Special attention should be paid to *Lipoptena fortisetosa*, an invasive species that continues to spread across Europe. *Lipoptena fortisetosa* is a blood-feeding ectoparasite that specifically targets cervids, including the sika deer (*Cervus nippon*), Siberian roe deer (*Capreolus pygargus*), and roe deer (*Capreolus capreolus*). This parasite also uses the red deer (*Cervus elaphus*), fallow deer (*Dama dama*), and moose (*Alces alces*) [5]. *Lipoptena fortisetosa* originates from eastern Siberia and the Far East [6]. The species was first described by Maa in Japan [6]. In Poland, *L. fortisetosa* was first identified in the region of Lower Silesia in the late 1980s, and its presence was confirmed in the Tatra Mountains and in northern Poland in 2007–2012 [5,7,8,9,10,11,12]. This ectoparasite was also isolated in the region of Wielkopolska in 2017 [13]. Deer keds target forest animals during host-seeking flights [14]. The duration of the host-seeking period is determined by the local microclimate and the insect’s phenology [14]. Louse flies shed wings when they find a definitive host. They are highly mobile on the host’s skin [15]. Deer keds reproduce by adenotrophic viviparity. The prevalence of *Lipoptena* spp. infestations in specific hosts ranges from 64% to 78%, depending on the species [16,17]. These ectoparasites were also identified in non-specific hosts. *Lipoptena fortisetosa* has been isolated from dogs [13]. Izdebska [18] confirmed the presence of *Lipoptena* spp. in bison (*Bison bonasus*). Deer keds were also found to attack cattle and horses [19,20]. Louse fly attacks and bites have also been reported in humans [21,22,23]. A single host can be infested by up to 16,000 insects [24]. Bites of the *Lipoptena* spp. may cause acute to chronic, eosinophilic to lymphocytic deer ked dermatitis [25]. Other infection symptoms include loss of hair, allergic rhinitis, conjunctivitis, or even anaphylactic shock [25,26,27,28].

Vectors are part of the One Health concept because they are responsible for the transmission of pathogens between wildlife, livestock, companion animals and humans [29,30]. Pathogens carried by arthropods cause vector-borne diseases. Vectors can carry viruses, bacteria, protozoa and helmints. Vector-borne viruses include, among others, the Chikungunya virus, bluetongue disease virus, dengue virus, Crimean-Congo hemorrhagic fever virus, West Nile virus, hantavirus, and Zika virus. The following bacteria are also transmitted by vectors: *Anaplasma* spp., *Bacillus* spp., *Bartonella* spp., *Borellia* spp., *Brucella* spp., *Clostridium* spp., *Coxiella* spp., *Erysipelothrix* spp., *Francisella* spp., *Leptospira* spp., *Listeria* spp., *Mycoplasma* spp., *Pasteurella* spp., *Rickettsia* spp., *Salmonella* spp., and *Yersinia* spp. Vectors also play a very important role in the life cycle of protozoa, including *Babesia* spp., *Leishmania* spp., *Plasmodium* spp., *Theileria* spp., and *Trypanosoma* spp. Similarly to other flies of the family Hippoboscidae [31], *L. fortisetosa* are potential vectors of infectious diseases [4,32]. DNA of *Coxiella* spp., *Theileria luwenshuni*, and *T. ovis* have been identified in *L. fortisetosa* in molecular analysis [33]. *Lipoptena fortisetosa* can also act as a vector for *Trypanosoma* spp. [34]. Trypanosome DNA was detected in 20% of *L. cervi* and in 48.64% of *L. fortisetosa* [35]. Deer keds harbored genetic material of *Anaplasma* spp. and *Rickettsia* spp. [36,37]. In isolated cases, DNA of *Borrelia* spp. was identified in louse flies [38,39]. The prevalence of *Bartonella* spp., which is transmitted vertically in deer keds, has been widely researched [40,41,42,43]. These pathogens’ DNA was found in up to 98% of louse flies [42,43].

The European population of *L. fortisetosa* continues to spread, and further research is needed to evaluate this ectoparasite’s role in the spread of infectious diseases. The expansion of the insect’s geographic range was confirmed by Italian and Estonian studies [44,45]. The existing research is largely based on single detections of the pathogen’s genetic material. Most of the research was conducted in small and limited locations. Louse flies’ impact on wildlife populations, livestock, companion animals and humans has not been fully elucidated. The identification of genetic material in these ectoparasites could contribute important information about pathogens.

The aim of this study was to identify pathogens with zoonotic potential (*A. ovis*, *A. phagocytophilum*, *Babesia* spp., *Bartonella* spp., *Borellia* spp., *Coxiella* spp., *Francisiella tularensis*, *Mycoplasma* spp., *Rickettsia* spp., and *Theileria* spp.) in *L. fortisetosa* infesting cervids and in host-seeking louse flies in the environment.

## 2. Results

**Figure 1:**
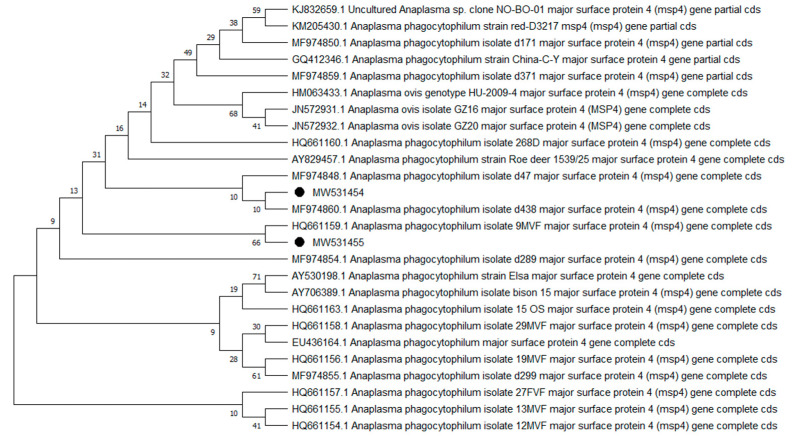
Phylogenetic topology for Neighbor Joining analysis of the MSP4 gene sequence of *Anaplasma phagocytophilum*. The unique haplotypes identified in this study are labeled with the corresponding sequence identification numbers and with dots. The reference sequences from GenBank are indicated in the tree. Bootstrap confidence values for branching reliability were calculated in 10,000 replicates.

**Figure 2:**
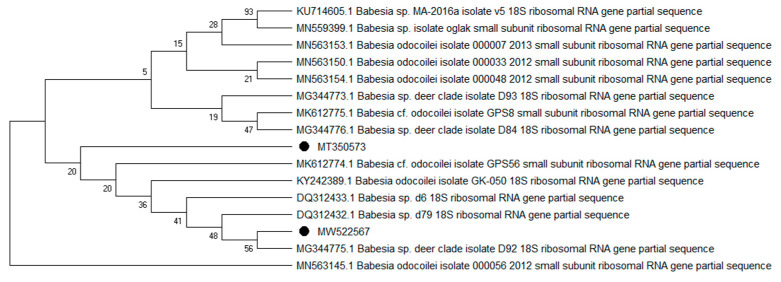
Phylogenetic topology for the Neighbor Joining analysis of the partial 18S rRNA gene sequence of *Babesia* spp. The unique haplotypes identified in this study are labeled with the corresponding sequence identification numbers and with dots. The reference sequences from GenBank are indicated in the tree. Bootstrap confidence values for branching reliability were calculated in 10,000 replicates.

**Figure 3:**
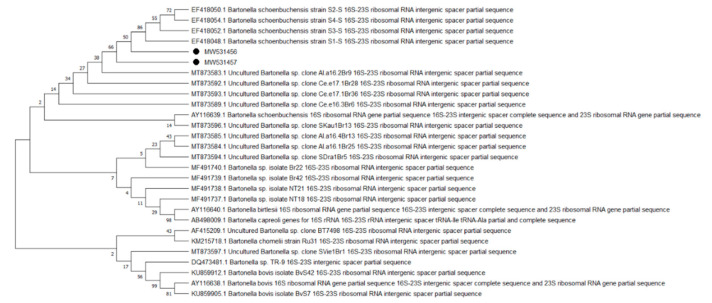
Phylogenetic topology for the Neighbor Joining analysis of the partial 16-23S gene sequence of *Bartonella* spp. The unique haplotypes identified in this study are labeled with the corresponding sequence identification numbers and with dots. The reference sequences from GenBank are indicated in the tree. Bootstrap confidence values for branching reliability were calculated in 10,000 replicates.

**Figure 4:**
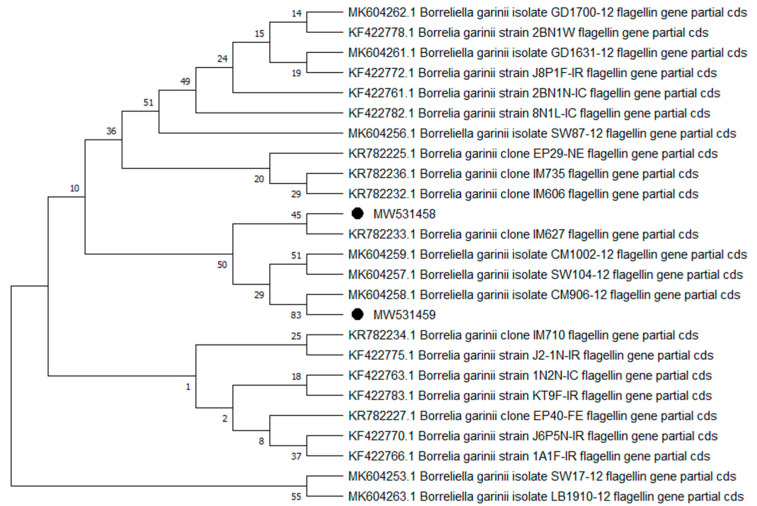
Phylogenetic topology for the Neighbor Joining analysis of the partial flagellin gene sequence of *Borrelia* spp. The unique haplotypes identified in this study are labeled with the corresponding sequence identification numbers and with dots. The reference sequences from GenBank are indicated in the tree. Bootstrap confidence values for branching reliability were calculated in 10,000 replicates.

**Figure 5:**
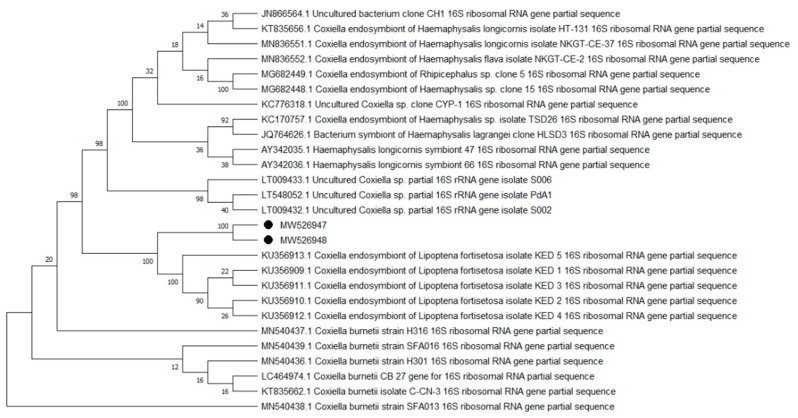
Phylogenetic topology for the Neighbor Joining analysis of the partial 16S rRNA gene sequence of *Coxiella* spp. The unique haplotypes identified in this study are labeled with the corresponding sequence identification numbers and with dots. The reference sequences from GenBank are indicated in the tree. Bootstrap confidence values for branching reliability were calculated in 10,000 replicates.

**Figure 6:**
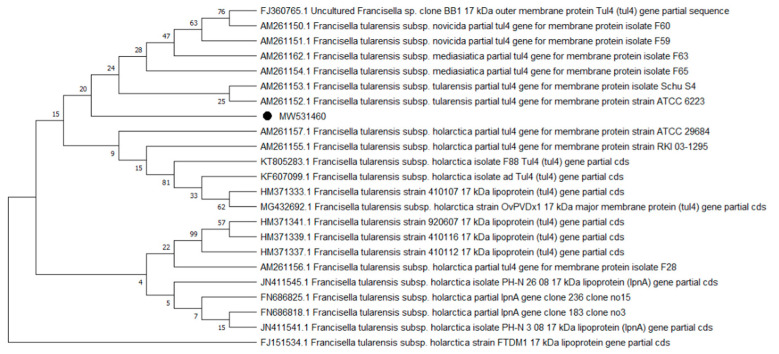
Phylogenetic topology for the Neighbor Joining analysis of the partial TUL4 gene sequence of *Francisella* spp. The unique haplotypes identified in this study are labeled with the corresponding sequence identification numbers and with dots. The reference sequences from GenBank are indicated in the tree. Bootstrap confidence values for branching reliability were calculated in 10,000 replicates.

**Figure 7:**
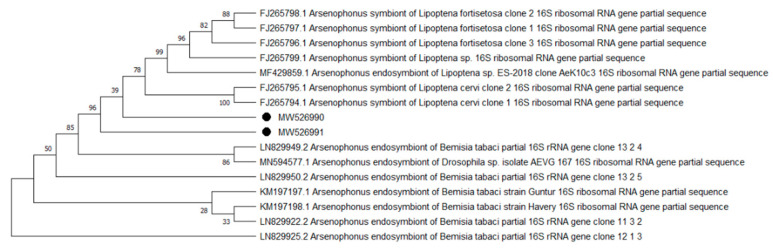
Phylogenetic topology for the Neighbor Joining analysis of the partial 16S rRNA gene sequence of *Arsenophonus* spp. The unique haplotypes identified in this study are labeled with the corresponding sequence identification numbers and with dots. The reference sequences from GenBank are indicated in the tree. Bootstrap confidence values for branching reliability were calculated in 10,000 replicates. The obtained sequences were acquired by cross-reaction.

**Figure 8:**
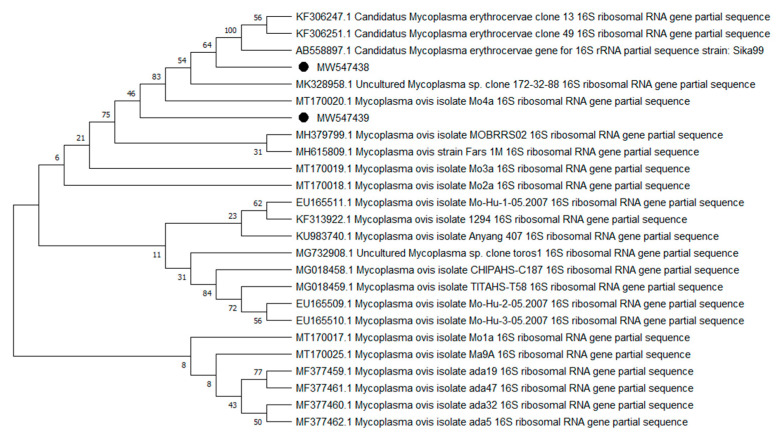
Phylogenetic topology for the Neighbor Joining analysis of the partial 16S rRNA gene sequence of *Mycoplasma* spp. The unique haplotypes identified in this study are labeled with the corresponding sequence identification numbers and with dots. The reference sequences from GenBank are indicated in the tree. Bootstrap confidence values for branching reliability were calculated in 10,000 replicates.

**Figure 9:**
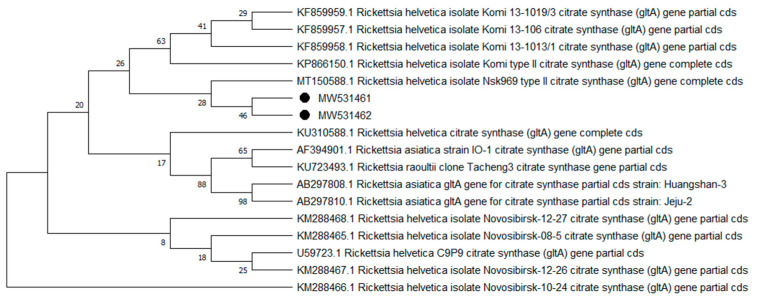
Phylogenetic topology for the Neighbor Joining analysis of the partial gltA gene sequence of *Rickettsia* spp. The unique haplotypes identified in this study are labeled with the corresponding sequence identification numbers and with dots. The reference sequences from GenBank are indicated in the tree. Bootstrap confidence values for branching reliability were calculated in 10,000 replicates.

**Figure 10:**
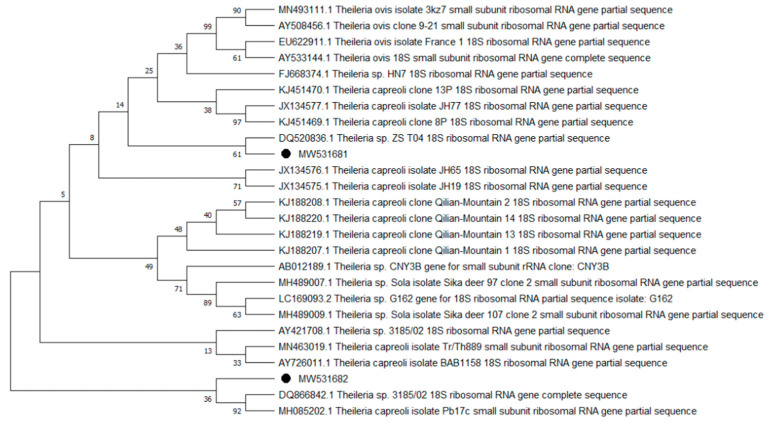
Phylogenetic topology for the Neighbor Joining analysis of the partial 18S rRNA gene sequence of *Theileria* spp. The unique haplotypes identified in this study are labeled with the corresponding sequence identification numbers and with dots. The reference sequences from GenBank are indicated in the tree. Bootstrap confidence values for branching reliability were calculated in 10,000 replicates.

Overall, 9 of the 10 analyzed pathogens’ DNA was identified, including 5 in group E and 9 in group A. In group A, *A. phagocytophilum* was identified in 20 (8%) samples, *Babesia* spp. —in 51 (20.4%) samples, *Bartonella* spp. —in 158 (63.2%) samples, *Borrelia* spp. —in 12 (4.8%) samples, *Coxiella* spp. —in 12 (4.8%) samples, *Francisella* spp. —in 7 (2.8%) samples, *Mycoplasma* spp. —in 74 (29.6%) samples, *Rickettsia* spp. —in 76 (30.4%) samples, and *Theileria* spp. —in 49 (19.6%) samples. In group E, *Bartonella* spp. was identified in 89 (35.6%) samples, *Coxiella* spp. —in 3 (1.2%) samples, *Francisella tularensis*, —in 2 (0.8%) samples, *Mycoplasma* spp. —in 17 (6.8%) samples, and *Rickettsia* spp. —in 30 (12%) samples. Detailed data are presented in Table 1. The representative sequences of selected pathogens were deposited in the GenBank database of the National Center for Biotechnology Information under the following accession numbers: *A. phagocytophilum* MW531454, MW531455; *Babesia* spp. MT350573, MW522567; *Bartonella* spp. MW531456, MW531457; *Borrelia* spp. MW531458, MW531459; *Coxiella* spp. MW526947, MW526948; *Francisella* spp. MW531460; *Mycoplasma* spp. MW547438, MW547439; *Rickettsia* spp. MW531461, MW531462; *Theileria* spp. MW531681, MW531682. The phylogenetic trees for the obtained sequences are presented in Figure 1, Figure 2, Figure 3, Figure 4, Figure 5, Figure 6, Figure 7, Figure 8, Figure 9 and Figure 10.

*Anaplasma phagocytophilum* sequences (MW531454, MW531455) were characterized by 99.76% similarity with sequences from Hungary (MF974860) and Slovakia (HQ661159). One sequence (MT350573) was identical with *Babesia odocoilei* from Norway (MK612774). One sequence (MW522567) was identical with *Babesia* spp. from the Czech Republic (MG344773). *Bartonella schoenbuchensis* sequences (MW531456, MW531457) were identical with sequences from France (AY116639) and Poland (EF418048, EF418052). *Coxiella* spp. sequences (MW531458, MW531459) were characterized by 97.63–97.64% similarity with sequences from South Korea (KU356909-KU356913). One sequence (MW531460) was identical with the *Francisella tularensis subsp. holarctica* sequence from Germany (EF418048, EF418052). The remaining sequences of *Francisella* spp. have been identified as *Arsenophonus* spp. One sequence (MW547438) was identical with the Candidatus *Mycoplasma erythrocervae* sequence from Japan (KF306251, KF306247, AB558897). Another *Mycoplasma* spp. sequence (MW547439) was identical with *Mycoplasma ovis* sequences, from Hungary (EU165509) and Turkey (MF377462). Sequences of *Rickettsia* spp. (MW531461, MW531462) were identical with *Rickettsia helvetica* sequences from Russia (KU310588) and France (U59723). One sequence (MW531681) was characterized by 99.73% similarity with the *Theileria capreoli* sequence from Turkey (MN463019). A second *Theileria* spp. sequence (MW531682) was identical with sequences from Turkey (MN463019) and Spain in red deer imported from Germany (AY421708).

Deer keds sampled from animals were significantly more likely to harbor pathogens than the insects from environmental samples. Detection of pathogens’ genetic material was significantly higher in female than male flies. DNA of *A. phagocyphilum* was more frequently identified in females than males. *Babesia* spp. genetic material was significantly more prevalent in *L. fortisetosa* sampled in the Warmia-Masuria and Lubusz voivodeships. The prevalence of *Bartonella* spp., *Coxiella* spp., *Mycoplasma* spp., and *Rickettsia* spp. DNA was significantly higher in group A than in group E. Genetic material of *Theileria* spp. was less frequently detected in insects sampled in the Lubusz voivodeship. Detailed data are presented in Table 2.

Significant relationships between the prevalence of the examined pathogens were observed. The values of Cramer’s V were indicative of the absence of associations or weak associations. The relationships between the prevalence of the analyzed pathogens are presented in detail in Table 3.

## 3. Discussion

The results of this study indicate that *L. fortisetosa* is ubiquitous in the analyzed voivodeships. In a previous study, this ectoparasite was identified only in isolated locations [5]. Deer keds could spread to new locations due to low level of host specificity as well as animal migrations. *Lipoptena fortisetosa* is an invasive species in Poland, but its population has been expanding steadily, which suggests that it has adapted well to the environmental conditions of Central Europe [14]. Louse flies infest hosts directly after pupation, and the emerged insects cover areas with a radius of 50 m [46]. Winged flies are responsible for the majority of non-specific host infestations, including in humans [23], companion animals [13], and livestock [19]. The host-seeking flights of *L. fortisetosa* may pose a threat for forest workers and hunters [23,47]. Hunters may become infested with *Lipoptena* spp. during the evisceration of wild animals. Hunting dogs may be attacked by these ectoparasites during tracking [13]. Cattle and horses grazing in the vicinity of forests also may be at risk of *L. fortisetosa* infestation [19,20]. People wearing dark clothing and animals with a dark fur coat are most susceptible to insect flights [48].

In this study, the prevalence and number of pathogens were lower in winged deer keds than in individuals sampled from cervids. Due to the fact that louse flies drop their wings upon attachment to the host, any subsequent switch in host is made more difficult [49]. However, *Lipoptena* spp. may switch hosts during the breeding season of cervids [50]. Infested mothers can also transmit these ectoparasites to their offspring [51]. In addition to insect bites, the infectious agent could also be transmitted when an insect is crushed on the skin [52]. Our results showed that *Lipoptena fortisetosa* carry DNA of pathogens, which might be collected through bloodmeal and transferred during the embryonic development of the larvae. The genetic material of *Babesia* spp., *Borrelia* spp., and *Theileria* spp. was detected only in deer keds that had direct contact with cervids. This is the first study to detect genetic material of *Babesia* spp. in deer keds. In addition, it is interesting to identify the DNA of *Borrelia* spp. in these ectoparasites in light of the fact that cervids are incompetent hosts for these bacteria [53]. The genetic material of *Bartonella* spp., *Mycoplasma* spp., and *Rickettsia* spp. was identified additionally in winged insects sampled from the environment. The presence of the wings means that these individuals had no previous contact with the host [54]. Some pathogens may be carried by vectors without direct contact with a host. This is generally the result of acquiring a blood meal by a female. Due to the characteristic development of the new generation, host seekers may still carry remnants of a previous blood meal.

Interestingly, the analyzed insects also harbored *Coxiella* spp. and *Francisella tularensis*, and the prevalence of these bacteria in deer keds has been poorly investigated to date. These bacterial species were identified in both insect groups; however, most of the obtained sequences were identified as endosymbionts. In addition, the present findings do not provide sufficient evidence to substantiate the above claim because the isolation kit used in this study is intended to detect trace amounts of DNA. Pupae developing in the forest understory could have been contaminated with environmental bacteria. *Coxiella* spp. and *Francisella* spp. colonize soil [55,56], where pupation occurs. Additionally, it is interesting to find the Candidatus *Mycoplasma erythrocervae* bacterium, which was most closely related to isolates from Japan [57]. Either the variance of this genetic material associated with the DNA fragment under test is low, or the pathogen has been introduced with sika deer (*Cervus nippon*). It is possible that with the introduction of this species to Poland, the pathogen expanded in the population of native cervids. The possible presence of another pathogen of Japanese origin (*Bartonella* spp.) was also suggested in other studies [43].

Our description of pathogens ‘genetic material detection with molecular tests might be an important consideration in research on deer keds’ role in the transmission of infectious diseases. This research allows for a preliminary determination of potential pathogens for which *L. fortisetosa* may serve as vector. However, the transmission of the infectious agents by *L. fortisetosa* should be evaluated with the use of Koch’s postulates [58,59,60]. For example, the development of *Babesia* spp. and *Theileria* spp. in *Lipoptena* spp. seems doubtful because ticks are the only known definitive hosts for these protozoa [61]. However, further research is needed to confirm this assumption. Despite the above, deer keds may act as mechanic vectors for these pathogens [4,37]. *Lipoptena cervi* has been suggested to mechanically transmit *A. phagocytophilum* to cervids [37,62]. Hornok et al. [63] identified *Theileria* spp. in stable flies (*Stomoxys calcitrans*) and suggested that this ectoparasite may pose a risk of mechanical transmission of theilerioses. Therefore, the research should assess whether a similar phenomenon may occur in deer keds. *Lipoptena fortisetosa* may be the potential biological vector for *Trypanosoma* spp. [35]. Currently, *Bartonella* spp. is the most comprehensively studied pathogen carried by *Lipoptena* spp., which appears to pose the greatest risk in infestation with these ectoparasites [40,41,42,43].

In the present study, genetic material of pathogens was more frequently identified in female than male *L. fortisetosa*, which could be explained by the fact that, in some ectoparasite species, females ingest more food than males [64,65]. Female insects of the genus *Lipoptena* spp. suck blood to draw nutrients that are needed for embryogenesis and intrauterine feeding of larvae [66]. Moreover, the exposure to microorganisms that are sucked with blood by females might last longer in the next generation. The logistic regression model revealed differences in pathogen prevalence between groups, which could suggest that some pathogen DNA may still be present in host-seeking ectoparasites. This study explored the idea that several of the detected microorganisms’ DNA might have been vertically transmitted. Some pathogens were also more prevalent in selected voivodeships. These differences can probably be attributed to variations in pathogen prevalence across cervid populations [67,68]. The calculated values of Cramer’s V point to the absence of significant associations between the prevalence of the studied pathogens, which could suggest that other vectors play a role in the transmission of these infectious agents.

In the future, deer keds could be used as biological markers of vector-borne diseases in cervid populations, due to the biology and behavior of these ectoparasites as well as specific ectoparasite-host relationships [46]. The acquisition of biological material—in particular, blood—that is suitable for molecular analyses could be problematic if the appropriate transportation and analytical equipment and facilities are not available. Alternatively, *Lipoptena* spp. can be obtained from hosts that are more accessible during field studies. The present study demonstrated that DNA of the infectious agents might be identified in deer keds. In order to fully assess the potential of these ectoparasites as biological markers, it is necessary to compare the results of molecular analyses examining the blood samples with insects collected from the same animal.

Our studies showed that deer keds may harbor pathogenic microorganisms of significance; however, further investigation is needed to confirm whether intact and viable pathogens are indeed present, and if so, whether they can be transmitted by deer keds. Experiments performed on laboratory animals, which have not been performed to date, would elucidate the role of *Lipoptena* spp. in the eventual transmission of vector-borne diseases. Future research should focus on methods of controlling these ectoparasites and protecting humans and animals against their attacks. Greater attention should also be paid to *L. fortisetosa* in Central Europe, because its impact on indigenous cervid populations remains largely unknown.

## 4. Materials and Methods

### 4.1. Sample Collection

**Figure 11:**
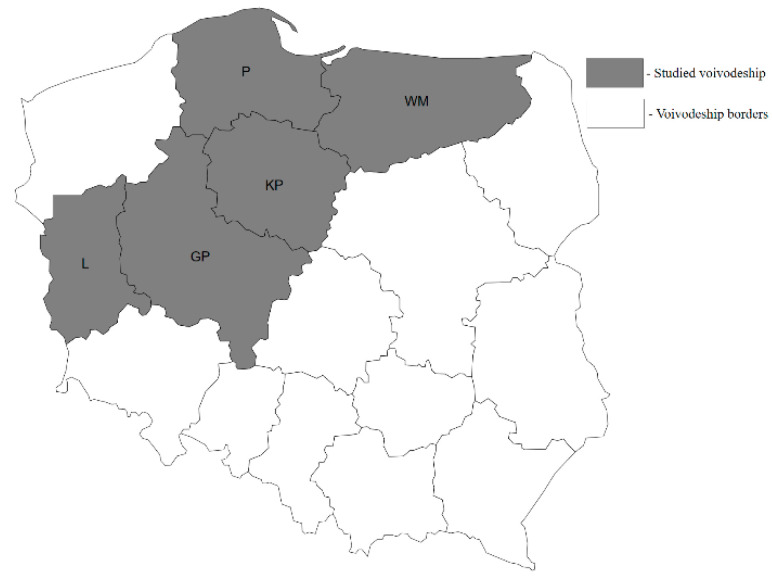
Map of Poland highlighting voivodships where *Lipoptena fortisetosa* samples were collected. Legend: GP—Greater Poland voivodeship; KP—Kuyavia-Pomerania voivodeship; L—Lubusz voivodeship; P—Pomerania voivodeship; WM—Warmia-Masuria voivodeship.

Deer keds were sampled in 2019 in five Polish voivodeships: Greater Poland, Kuyavia-Pomerania, Lubusz, Pomerania, and Warmia-Masuria. The location of these voivodeships on a map of Poland is presented in Figure 11. The collected specimens of *L. fortisetosa* were divided into two groups: Animals (A)—insects sampled from cervids, and Environment (E)—host-seeking insects sampled from the environment. A total of 500 *L. fortisetosa* individuals were sampled for the study. Group A was composed of 250 deer keds. Insects were collected from the bodies of dead or living cervids. One ectoparasite from each animal was randomly selected for analysis. Fifty individuals were acquired from each studied voivodeship. Group E consisted of 250 deer keds. Insects were collected in randomly selected Central European mixed forests. Up to 5 individuals of *L. fortisetosa* were acquired from each site. The investigator walked through the forest in the vicinity of wild animal habitats, wearing brown cotton clothing. Insects were captured after landing on the clothing and were immediately placed in test tubes. Fifty insects were obtained from each examined voivodeship. Group A and E ectoparasites were immersed in 70% ethanol in test tubes.

### 4.2. Species Identification

The sampled insects were analyzed in the Biological Hazard Laboratory at the Faculty of Veterinary Medicine of the University of Warmia and Mazury in Olsztyn. The species and sex of *L. fortisetosa* were identified based on the number of erect hairs on the mesonotum, the length and structure of palpi, wing venation, and body dimensions [3,44]. Images were acquired under the Leica M165C stereoscopic microscope (Leica, Wetzlar, Germany) with the use of Leica Application Suite 4.4 software (Leica, Wetzlar, Germany). Samples containing different insect species were excluded from further analysis.

### 4.3. DNA Extraction

*Lipoptena fortisetosa* were removed from test tubes, dried at room temperature for 15 min, and crushed individually with a sterile glass rod in sterile test tubes. Genomic DNA was extracted from each sample with the Sherlock AX universal kit (A&A Biotechnology, Gdynia, Poland) according to the manufacturer’s instructions. DNA was eluted in 40 µl of TE buffer. The concentration of the final product was determined with the Nano Drop 2000 spectrophotometer (Thermo Fisher Scientific, Waltham, USA). The extracted DNA was stored at −20 °C until analysis.

### 4.4. Selection of Pathogens for Analysis

Based on a review of the literature [67,68,69,70,71,72,73,74,75,76,77,78,79,80], the following pathogens were selected and identified: *Anaplasma ovis*, *A. phagocytophilum*, *Babesia* spp., *Bartonella* spp., *Borellia* spp., *Coxiella burnetii*, *Francisiella tularensis*, *Mycoplasma* spp., *Rickettsia* spp., and *Theileria* spp.

### 4.5. Polymerase Chain Reaction

Primer sequences and PCR conditions are presented in Table 4. Every reaction was carried out in a final volume of 25 µL, containing 2.5 µL of 10× Standard Taq Reaction Buffer (Biolabs, Boston, MA, USA, USA), 0.5 μL of 10 mM dNTPs (Biolabs, USA), 0.5 μL of 10 μM solution of each primer, 1 μL of extracted DNA, 0.125 µL of Taq DNA polymerase (Biolabs, USA), and 19.875 µL of double-distilled water. The reaction was carried out in the Veriti Thermal Cycler (Applied Biosystems, Foster City, CA, USA). DNA was replaced with double-distilled water in the negative sample. The positive sample consisted of DNA samples of each analyzed pathogen and *L. fortisetosa* collected in a previous study. PCR products were electrophoresed on 1.5–2.5% agarose gel using a 100 bp DNA ladder as a molecular-weight size marker, stained with ethidium bromide, and viewed under a UV transilluminator. The two strongest expressed bands in each studied pathogen were sequenced. All PCR products that were DNA positive for *Francisella* spp. were validated using sequencing.

### 4.6. Sequencing

DNA samples were purified by ethanol precipitation. Cycle-sequencing reactions were carried out with the use of the described primers, BigDye Terminator Cycle Sequencing Kit (Applied Biosystems, Foster City, CA, USA), and the ABI PRISM 3100 Genetic Analyzer (Applied Biosystems, Foster City, CA, USA). The obtained nucleotide sequences were edited in the BioEdit program [91] and compared with GenBank data in the BLAST-NCBI program. A phylogenetic analysis of the obtained sequences and the corresponding GenBank sequences was conducted by the Neighbor Joining estimation in MEGA 10.1.17 [92]. Bootstrap confidence values for estimating branching reliability were calculated in 10,000 replicates.

### 4.7. Statistical Analysis

The significance of possible relationships between the presence of pathogens and sample characteristics was determined in a logistic regression model, where the dependent variable was the dichotomous variable (absence (0)/presence (1) of the analyzed pathogen), whereas the insects’ sex (male/female), group (A or E), and site (examined voivodeships) were the independent variables. The associations between the prevalence of the analyzed pathogens were examined by calculating Cramer’s V, where values close to 0 denoted weak associations and values approximating +1/−1 denoted stronger associations. Data were processed statistically in the Statistica 13.3 program (TIBCO Software Inc., Palo Alto, Santa Clara, CA, USA).

## 5. Conclusions

The analyzed deer ked samples, obtained from animals and the environment, harbored the genetic material of *A. phagocytophilum*, *Babesia* spp., *Bartonella* spp., *Borellia* spp., *Coxiella*-like endosymbionts, *Francisiella tularensis*, *Mycoplasma* spp., *Rickettsia* spp., and *Theileria* spp. Deer keds may be used as biological markers for identifying pathogens in cervid populations, but further research is needed to confirm this assumption. Future research might reveal the competency of the *L. fortisetosa* as a biological or mechanical vector. The role of *L. fortisetosa* as a vector of pathogens requires further, more extensive investigations due to their new colonization sites and attacks on humans and animals. These are important considerations that require further attention.

## Figures and Tables

**Table 1:** Prevalence of selected pathogens in *Lipoptena fortisetosa* with a division into voivodeships, groups, and sexes.

Species	Group A		Group E		Total n-500
	n-250		n-250		
	M	F	M	F	
	n-99	n-151	n-115	n-135	
*Anaplasma ovis*	0 (0%)	0 (0%)	0 (0%)	0 (0%)	0 (0%)
*Anaplasma phagocytophilum*	6 (6.0%)	14 (9.3%)	0 (0%)	0 (0%)	20 (4%)
*Babesia* spp.	21 (21.21%)	30 (19.86%)	0 (0%)	0 (0%)	51 (10.2%)
*Bartonella* spp.	64 (64.65%)	94 (62.25%)	37 (32.17)	52 (38.52%)	247 (49.4%)
*Borrelia* spp.	3 (3.03%)	9 (6.6%)	0 (0%)	0 (0%)	12 (24.30%)
*Coxiella* spp.	3 (3.03%)	9 (6.6%)	1 (2.87%)	2 (1.48%)	15 (3%)
*Francisella* spp.	2 (2.02%)	5 (3.31%)	0 (0%)	2 (1.48%)	9 (1.8%)
*Mycoplasma* spp.	22 (22.22%)	52 (34.44%)	9 (7.82%)	8 (5.93%)	91 (18.2%)
*Rickettsia* spp.	30 (30.30%)	46 (30.46%)	14 (12.17%)	16 (3.2%)	106 (21.2%)
*Theileria* spp.	22 (22.22%)	27 (17.88%)	0 (0%)	0 (0%)	49 (9.8%)

Legend: A—Animals group; E—Environment group; M—male; F—female.

**Table 2:** Logistic regression model presenting statistically significant relationships between the analyzed pathogens vs. the sex, group, and sampling site of deer keds.

Parasite Species (Dependent Variables)	*p*-Value (for the Model)	Independent Variables		*SE*	*W*	*p*-Value *	*OR*
Total number	<0.001	sex	M	0.32	4.06	0.044	0.584
			F				1.476
		group	A	0.22	92.32	<0.001	8.04
			E				0.12
*Anaplasma phagocytophilum*		sex	M	0.56	2.85	0.03	1.24
			F				0.88
*Babesia* spp.		site	WM	0.54	4.33	0.037	3.09
			L	0.55	3.67	0.045	2.85
*Bartonella* spp.		group	A	0.20	26.04	<0.001	2.803
			E				0.36
*Coxiella* spp.		group	A	0.582	4.445	0.035	3.410
			E				0.293
*Mycoplasma* spp.		group	A	0.283	35.53	<0.001	5.40
			E				0.19
*Rickettsia* spp.		group	A	0.226	16.559	<0.001	2.51
			E				0.40
*Theileria* spp.		site	L	0.653	6.483	0.011	0.19

Legend: *—*p*-value < 0.05; SE—standard error; W—Wald coefficient; OR—odds ratio; M—male; F—female; A—animals group; E—environment group; WM—Warmia-Masuria; L—Lubusz.

**Table 3:** Associations between the prevalence of the analyzed pathogens in *Lipoptena fortisetosa*, based on the calculated values of Cramer’s V.

Species	*Anaplasma phagocytophilum*	*Babesia* spp.	*Bartonella* spp.	*Borrelia* spp.	*Coxiella* spp.	*Francisella tularensis*	*Mycoplasma* spp.	*Rickettsia* spp.	*Theileria* spp.
*Anaplasma phagocytophilum*	-	0.033 ^b^	0.013 ^b^	0.21 *^,a^	0.20 *^,a^	0.057 ^b^	0.062 ^b^	0.035 ^b^	0.040 ^b^
*Babesia* spp.	0.079 ^a^	-	0.024 ^b^	0.026 ^b^	0.029 ^b^	0.11 ^b^	0.041 ^b^	0.011 ^b^	0.050 ^b^
*Bartonella* spp.	0.026 ^a^	0.094 *^,a^	-	0.005 ^b^	0.016 ^b^	0.037 ^b^	0.029 ^b^	0.009 ^b^	0.04 ^b^
*Borrelia* spp.	0.21 *^,a^	0.077 ^a^	0.032 ^a^	-	0.032 ^b^	0.043 ^b^	0.1 ^b^	0.027 ^b^	0.078 ^b^
*Coxiella* spp.	0.16 *^,a^	0.010 ^a^	0.037 ^a^	0.043 ^a^	-	0.045 ^b^	0.073 ^b^	0.080 ^b^	0.025 ^b^
*Francisella* spp.	0.035 ^a^	0.12 *^,a^	0.024 ^a^	0.025 ^a^	0.30 ^a^	-	0.063 ^b^	0.059 ^b^	0.041 ^b^
*Mycoplasma* spp.	0.01 ^a^	0.13 *^,a^	0.028 ^a^	0.128 *^,a^	0.032 ^a^	0.13 ^a^	-	0.028 ^b^	0.055 ^b^
*Rickettsia* spp.	0.01 ^a^	0.069 ^a^	0.077 ^a^	0.0080 ^a^	0.11 *^,a^	0.039 ^a^	0.050 ^a^	-	0.046 ^b^
*Theileria* spp.	0.08 *^,a^	0.16 *^,a^	0.11 ^a^	0.12 *^,a^	0.012 ^a^	0.008 ^a^	0.052 ^a^	0.090 ^a^	-

Legend: *—*χ^2^ p*-value < 0.05; ^a^—Cramer’s V for all samples; ^b^—Cramer’s V for animals group samples.

**Table 4:** Primers and PCR conditions.

Species	Target Gene	Name of Primer	Primer Sequence (5′–3′)	Expected Size (bp)	PCR Cycle Conditions	Reference
*Anaplasma ovis*	MSP4	MSP43R	CCG GAT CCT TAG CTG AAC AGG AAT CTT GC	347	94 °C/5 min; 40 cycles: 94 °C/30 s, 60 °C/30 s, 72 °C/1 min; 72 °C/7 min	Ochirkhuu et al. [81]
		MSP45F	GGG AGC TCC TAT GAA TTA CAG AGA ATT GTT TAC			
*Anaplasma phagocytophilum*	MSP4	Aphamsp4F	ATGAATTACAGAGAATTGCTTGTAGG	849	94 °C/5 min; 40 cycles: 94 °C/10 s, 58 °C/10 s, 72 °C/50 s; 72 °C/5 min	Bown et al. [82]
		Aphamsp4R	TTAATTGAAAGCAAATCTTGCTCCTATG			
Bovine * *Babesia* spp.	18S rRNA	BabsppF1	GTTTCTGMCCCATCAGCTTGAC	440	45 cycles: 94 °C/30 s, 61 °C/30 s, 72 °C/45 s; 72 °C/10 min	Hilpertshauser et al. [83]
		BabsppR	CAAGACAAAAGTCTGCTTGAAAC			
*Bartonella* spp.	16–23S gene	Bartonella spp. F	(C/T)CTTCGTTTCTCTTTCTTCA	154-260	95 °C/2min; 45 cycles: 95 °C/1 min, 60 °C/1 min, 72 °C/30 s; 72 °C/5 min	Jensen et al. [84]
		Bartonella spp. R	AACCAACTGAGCTACAAGCC			
*Borrelia* spp.	flagellin	Borrelia spp. flaF	ACATATTCAGATGCAGACAGAGGT	350	95 °C/5 min; 40 cycles: 95 °C/1 min, 55 °C/1 min, 72 °C/30 s; 72 °C/5 min	Barbour et al. [85]
		Borrelia spp. flaR	AACAGCTGAAGAGCTTGGAATG			
*Coxiella burnetii*	16S rRNA	Coxiella 16SrRNA F	ATTGAAGAGTTTGATTCTGG	1457	95 °C/3 m; (2 cycles: 95 °C/30 s, 58 °C/30 s, 72 °C/2 min; 10 cycles: 95 °C/30 s, 58–50 °C/30 s (–2 every 2 cycles), 72 °C/2 min; 30 cycles: 95 °C/30 s, 48 °C/30 s, 72 °C/2 min); 72 °C/5 min	Zheng et al. [86]
		Coxiella 16SrRNA R	CGGCTTCCCGAAGGTTAG			
*Francisella tularensis*	TUL4	Ff393	ATGGCGAGTGATACTGCTTG	248	98 °C/30 s; 35 cycles: 98 °C/10 s, 53.4 °C/ 30 s, 72 °C/1 min; 72 °C/10 min	Long et al. [87]
		Ff642	GCATCATCAGAGCCACCTAA			
*Mycoplasma* spp.	16S rRNA	HBT-F	ATA CGG CCC ATA TTC CTA CG	595	94 °C/10 min; 40 cycles: 95 °C/30 s, 60 °C/30 s, 72 °C/30 s; 72 °C/10 min	Liu et al. [88]
		HBT-R	TGC TCC ACC ACT TGT TCA			
*Rickettsia* spp.	gltA	Rickettsia gltA F	GCAAGTATCGGTGAGGATGTAAT	401	95 °C/3 min; 40 cycles: 95 °C/15 s, 48 °C/30 s, 72 °C/30 s; 72 °C/7 min	Labruna et al. [89]
		Rickettsia gltA R	GCTTCCTTAAAATTCAATAAATCAGGAT			
*Theileria* spp.	18S rRNA	989	AGT TTC TGA CCT ATC AG	1098	94 °C/5 min; 35 cycles: 94 °C/30 s, 53 °C/30 s, 72 °C/1 min; 72 °C/7 min	Li et al. [90]
		990	TTG CCT TAA ACT TCC TTG			

Legend: *—B. divergens, B. bigemina, B. major.

## Data Availability

The original contributions presented in the study are included in the article. Further inquiries can be directed to the corresponding author.
